# Paradoxical Embolization of the Bilateral Subclavian Arteries After High Tibial Osteotomy

**DOI:** 10.5435/JAAOSGlobal-D-19-00044

**Published:** 2019-08-06

**Authors:** Takahito Miyake, Osamu Obayashi, Akio Kanda, Hideshi Okada, Shinji Ogura, Kazuo Kaneko

**Affiliations:** From the Advanced Critical Care Center, Gifu University Hospital, Gifu-shi, Gifu, Japan (Dr. Miyake, Dr. Okada, and Dr. Ogura); the Department of Orthopaedic Surgery, Juntendo Shizuoka Hospital, Izunokuni, Shizuoka, Japan (Dr. Miyake, Dr. Obayashi, and Dr. Kanda); and the Department of Orthopaedic Surgery, Juntendo University, Bunkyo-ku, Tokyo, Japan (Dr. Kaneko).

## Abstract

A patent foramen ovale provides a portal through which a thrombus might pass from the right side of the circulation to the left. A 65-year-old man underwent high tibial osteotomy after the diagnosis of the right knee osteoarthritis. On postoperative day 12, he developed bilateral arm paresthesia. Enhanced CT revealed emboli in the bilateral pulmonary and subclavian arteries and deep vein thrombosis in the left lower limb. Transesophageal echocardiography after treatment revealed a patent foramen ovale during the Valsalva maneuver. It was thought that bilateral arm paresthesias were caused by the arterial emboli in the bilateral subclavian arteries.

A patent foramen ovale (PFO) is a hemodynamically insignificant interatrial communication present in >25% of the adult cohort.^1^ Paradoxical emboli are very rare and represent fewer than 2% of all arterial emboli.^2^ Some reports of paradoxical cerebral emboli have been found, but few occurred in supra-aortic arteries.^2–4^ Here, we report a rare case of a massive paradoxical embolism in the bilateral supra-aortic arteries after knee surgery.

## Case Report

**Figure 1:**
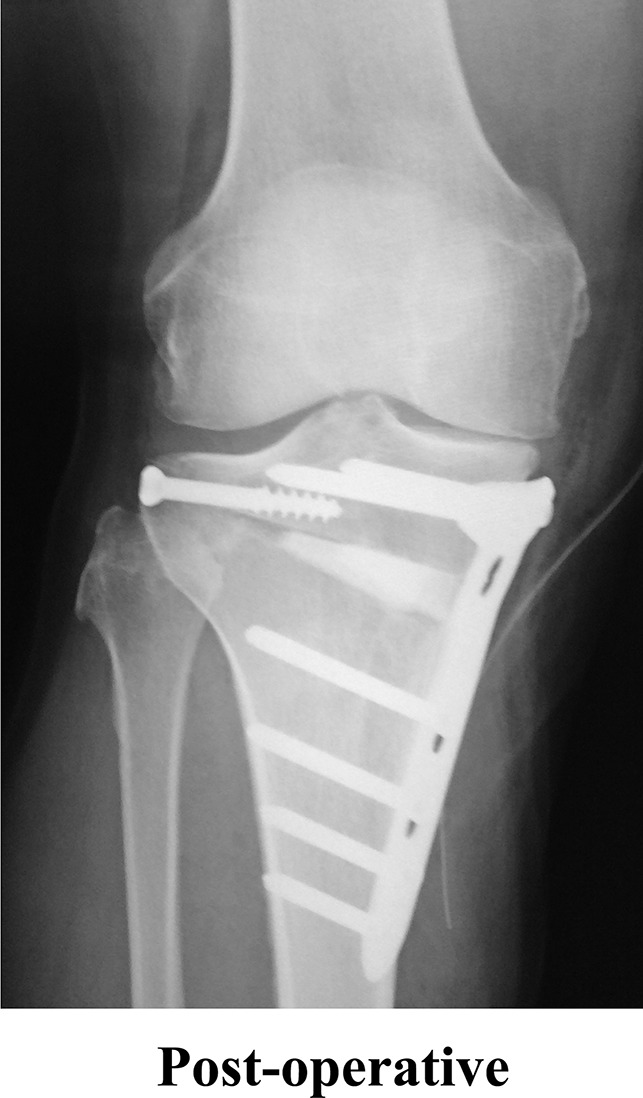
Right knee radiographs after surgery.

A 65-year-old man was admitted to our hospital for elective high tibial osteotomy after the diagnosis of right knee osteoarthritis (Figure 1). No other medical history was found. Surgery was performed under general and spinal anesthesia the day after admission. Intraoperatively, the medial osteotomy wedge was opened to 20°, and the desired correction was achieved. Postoperatively, non–weight-bearing mobilization was advised during 4-week rehabilitation. The patient was permitted to walk on crutches and was not required to take anticoagulants.

**Figure 2:**
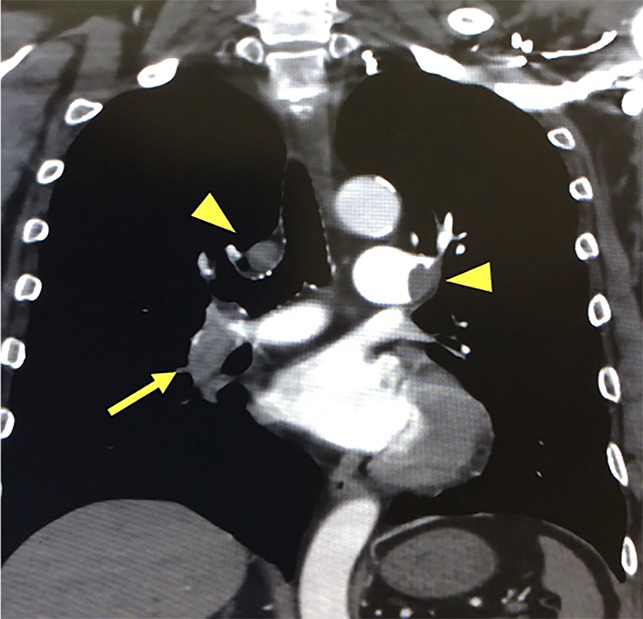
Enhanced CT images demonstrating embolization. Arrowheads: emboli in the bilateral subclavian arteries, arrow: embolus in the right pulmonary artery.

**Figure 3:**
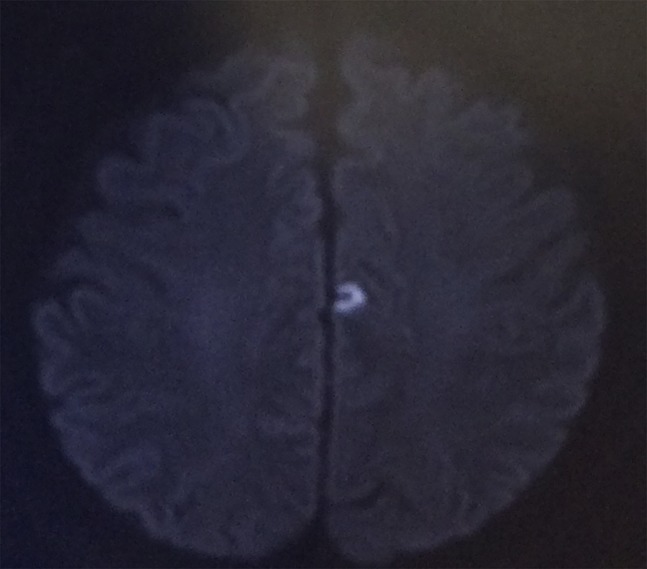
MRI diffusion image of the brain.

On postoperative day 12, he developed bilateral arm paresthesia. Urgent brain CT was performed, and no evidence of stroke was found. The patient did not appear to be dyspneic. Symptoms persisted until the next day, and reexamination was performed. The patient's oxygen saturation was 89% on room air, with normal breath sounds. His heart rate was 110 beats per minute. Blood pressure was not obtainable in either upper limb and was 130/80 mmHg in the lower limbs. The radial artery pulse was not palpable. Electrocardiogram revealed sinus tachycardia. Enhanced CT revealed emboli in the bilateral pulmonary and subclavian arteries and deep vein thrombosis (DVT) in the left lower limb (Figure 2). In addition, magnetic resonance imaging revealed a tiny brain infarction (Figure 3).

Physical examination and diagnostic imaging suggested that the patient's bilateral arm paresthesias were caused by arterial emboli in the bilateral subclavian arteries. An inferior vena cava filter device was inserted to prevent future emboli, and anticoagulation with intravenous heparin and urokinase was initiated. The levels of proteins C and S, antithrombin III, factor V Leiden, lupus anticoagulant, cardiolipin antibody immunoglobulins, prothrombin, and homocysteine were examined to assess the possibility of a hypercoagulable state, but all were normal. Intravenous anticoagulation was continued for 10 days with urokinase and 12 days with heparin, based on the follow-up results of enhanced CT. A direct oral anticoagulant was initiated when all intravenous drugs were discontinued.

On postoperative day 30, transesophageal echocardiography revealed a PFO during the Valsalva maneuver. On the following day, CT showed no arterial or venous emboli, and bilateral radial pulses could be palpated, and symptoms had completely resolved. The patient recovered uneventfully and was discharged from our hospital on postoperative day 44.

## Discussion

Paradoxical embolism is defined as a systemic embolism of venous or right atrial origin that passes through a right-to-left cardiac shunt. In the present case, the conduit for paradoxical embolism was a PFO. Although such emboli usually cannot travel from veins to arteries through the PFO because of relatively higher arterial pressures, Kollar et al^5^ indicated that in the event of a pulmonary embolism with a concurrent PFO, a sudden increase in right ventricular pressure can cause right-to-left arterial shunting. We consider that in our case, the emboli in the brain and bilateral subclavian arteries originated from a DVT, and the pulmonary embolism increased right ventricular pressure, causing right-to-left arterial shunting.

Ueno et al^6^ prospectively enrolled 240 consecutive patients with acute ischemic stroke and determined that 5% of the patients had a definite right-to-left shunt based on diagnostic criteria. Another study found that the diagnosis of paradoxical embolism was often difficult because signs of a DVT were absent in most patients.^4^ In addition, when patients reported of upper limb paresthesias, arterial embolism was not the initial diagnosis. Therefore, diagnosis of paradoxical embolism was difficult. Bedeir^3^ suggested that unexplained systemic emboli are caused by thrombi originating in the right side of the circulation, especially in the postoperative setting.

Few reports of paradoxical embolism are found in a supra-aortic artery, as occurred in this case, although some studies described paradoxical cerebral emboli.^2–4^ Although arterial thrombi are retrieved surgically in all cases, the optimal treatment of pulmonary and arterial emboli is not always clear because numerous therapeutic options are found, including anticoagulation, thrombolysis, mechanical thromboembolectomy, insertion of an inferior vena cava filter, and closure of the PFO. Considering the possibility of paradoxical embolism as a complication of knee surgery is important. In some reports, the incidence of a DVT was 28.1% to 41%.^6^ In addition, considering anticoagulants immediately after these surgeries is essential to prevent this complication.

## Conclusion

The present case showed bilateral emboli in the subclavian arteries and pulmonary embolism after knee surgery. These findings indicate that considering the possibility of coincidental paradoxical embolism after orthopaedic surgery is important.
